# The cerebral neurovascular coupling and glymphatic circulation dysfunction in children with attention deficit/hyperactivity disorder

**DOI:** 10.1186/s12888-026-08233-4

**Published:** 2026-05-29

**Authors:** Rui Hu, Fan Tan, Caohui Duan, Ling Sang, Chun Yang, Yong Wu, Yuhan Jiang, Yanwei Miao, Wen Chen, Xin Lou

**Affiliations:** 1 Department of Radiology, Taihe Hospital, Hubei University of Medicine, No. 32 South Renmin Road, Shiyan, Hubei Province China; 2 Department of Radiology, First Affiliated Hospital of Dalian Medical University, No. 222 Zhongshan Road, Xigang District, Dalian, Liaoning Province China; 3 Department of Radiology, The First Medical Center of Chinese PLA General Hospital, Beijing, China; 4 Department of Paediatrics, Taihe Hospital, Hubei University of Medicine, Shiyan, China

**Keywords:** Attention-deficit hyperactivity disorder, Neurovascular coupling, Along the perivascular space

## Abstract

**Background:**

Based on the executive dysfunction hypothesis, the deficiency of executive function is the basis of the core symptoms of attention deficit/hyperactivity disorder (ADHD). This study aims to apply MRI to evaluate changes in neurovascular coupling (NVC) and glymphatic system functions in patients with ADHD and to explore the relationship between these changes and executive function, in order to reveal the potential neural mechanisms underlying ADHD symptomatology.

**Methods:**

This study enrolled sixty-six individuals diagnosed with ADHD and forty-one healthy control participants (HC). We compared the differences in NVC and the index along the perivascular space (ALPS) between the two groups. Partial correlation analysis was employed to examine the associations among clinical data, executive function, the ALPS index, and NVC alterations in ADHD patients. Furthermore, a combined diagnostic model was developed using NVC, the ALPS index, and sleep duration, and its diagnostic performance was assessed through logistic regression analysis. The robustness of the joint diagnostic model was subsequently evaluated using a five-fold cross-validation approach.

**Results:**

In comparison to the HC group, patients with ADHD exhibited a significant reduction in NVC and alterations in the ALPS. Specifically, the ADHD group demonstrated decreased NVC across 19 brain regions, encompassing 8 regions within the frontal lobe, 5 within the parietal lobe, 4 within the occipital lobe, and 2 within the temporal lobe. Partial correlation analysis revealed an association between NVC values and the ALPS index, executive function, and sleep duration. Furthermore, receiver operating characteristic (ROC) analysis demonstrated good diagnostic efficacy of the combined model, validated through five-fold cross-validation, yielding an area under the curve (AUC) of 0.863, 95% CI: (0.790, 0.926), with a sensitivity of 0.864 and a specificity of 0.683.

**Conclusion:**

Children with ADHD exhibit impairments in the NVC and the glymphatic system function, with NVC dysfunction linked to glymphatic impairment and executive function deficits. This study offers novel insights into ADHD-related brain dysfunction through the perspectives of NVC and the glymphatic system.

**Clinical trial number:**

Not applicable.

**Graphical Abstract:**

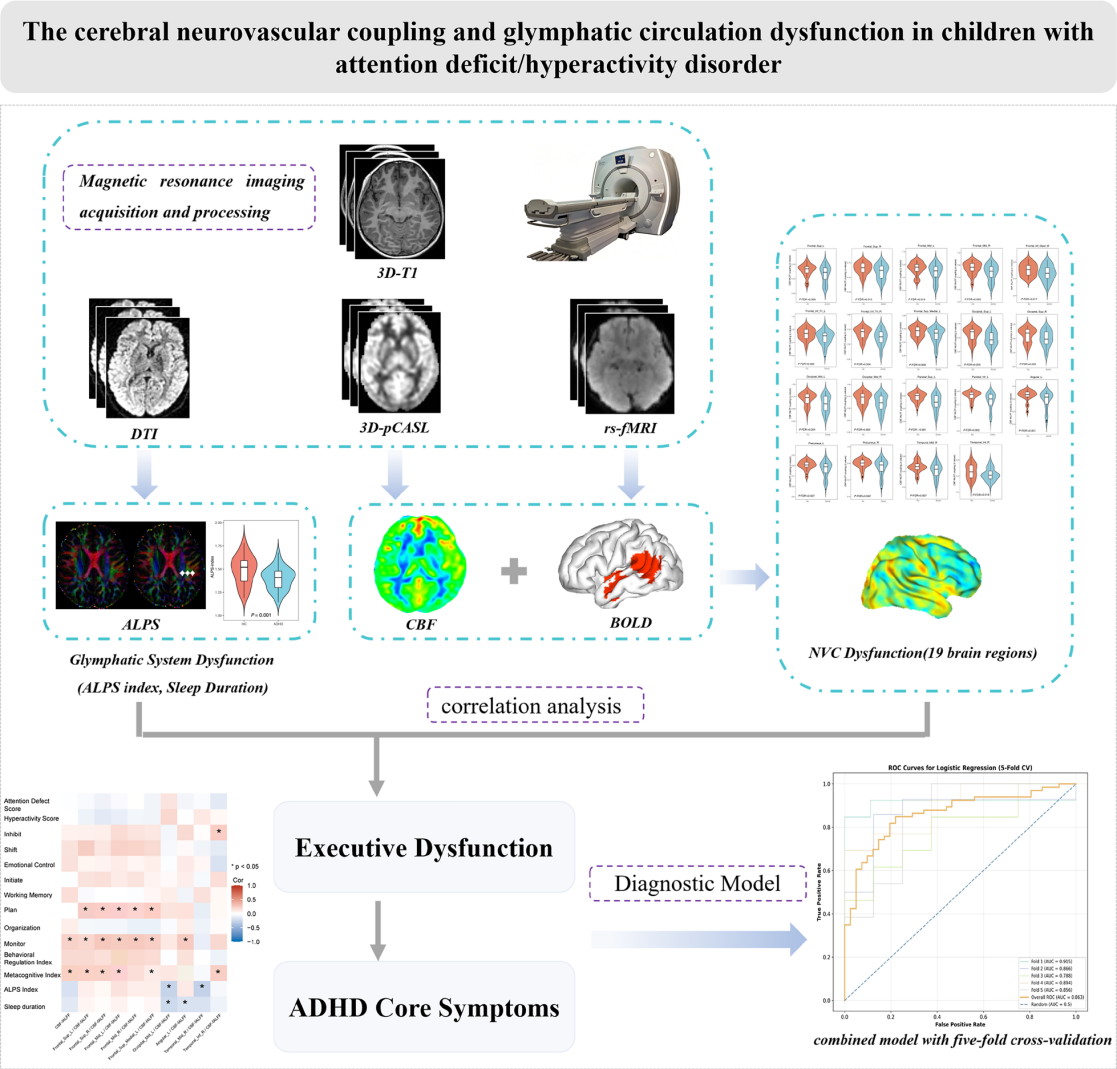

## Introduction

Attention deficit/hyperactivity disorder (ADHD), a chronic neurodevelopmental disorder, is characterized by persistent inattention, hyperactivity, and impulsivity [1]. Currently, the pathogenesis of ADHD remains poorly understood. It is worth noting that deficits in executive function are considered one of the core features of ADHD and can be observed across patients of all age groups [2–4]. Therefore, it is essential to conduct a comprehensive evaluation of executive function in patients with ADHD and to investigate potential neuroimaging markers associated with these cognitive impairments.

An increasing body of evidence has revealed the connection between sleep, ADHD, and executive function [5–8]. One of the key mechanisms may involve the promoting effects of sleep on the function of the Glymphatic System. This system mediates the exchange of cerebrospinal fluid and interstitial fluid through perivascular pathways and is a core mechanism for removing metabolic waste from the brain [9–13]. The diffusion tensor image-analysis along the perivascular space (DTI-ALPS) method provides an indirect and non-invasive means of evaluating the activity of the glymphatic system in individuals [14]. The functional clearance efficiency of the glymphatic system highly depends on the normal operation of Neurovascular Coupling (NVC). NVC refers to the precisely matched functional and spatial relationship between neural activity and changes in local Cerebral Blood Flow (CBF), and this coupling mechanism is key to maintaining homeostasis in the brain [15, 16]. On the one hand, neural activity triggers an increase in local CBF, providing sufficient oxygen and energy substrates for active brain regions; on the other hand, the increase in blood flow also drives the flow of cerebrospinal fluid/interstitial fluid, accelerating the clearance of metabolic waste by the glymphatic system [17]. Therefore, any abnormality in NVC function may directly or indirectly impair the clearance efficiency of the glymphatic system.

Previous studies have successfully evaluated the status and abnormalities of NVC in healthy individuals [18] and in diseases such as hypertension, schizophrenia [19], and Alzheimer’s disease [20, 21] by using resting-state functional magnetic resonance imaging (rs-fMRI) combined with three-dimensional pseudo-continuous arterial spin labeling (3D-pCASL) technology [22]. However, the functional status and integrity of NVC in patients with ADHD have not been systematically studied. Moreover, the potential role of the glymphatic system, which is critical for clearing metabolic waste and is closely linked to NVC function, has never been examined in ADHD. Thus, the present study addresses this gap by simultaneously investigating NVC and glymphatic function in the same ADHD cohort. Accordingly, the primary research question of this study is: whether children with ADHD show altered NVC and glymphatic function compared to typically developing controls. The secondary research question is: whether NVC is associated with glymphatic function and core ADHD symptoms.

NVC can accelerate the inflow and clearance of the glymphatic system [16]. We speculate that changes in NVC function may indirectly affect the glymphatic system, and this may play an important role in the occurrence and development of ADHD. Therefore, examining both systems in ADHD provides a more integrated view of brain homeostasis than either system alone.

We hypothesized that children with ADHD exhibit abnormal NVC and glymphatic function. Additionally, impaired NVC function is linked to the glymphatic system dysfunction and core clinical manifestations of ADHD, particularly deficits in executive functioning. In summary, this study intends to combine 3D-pCASL and rs-fMRI to investigate the NVC status of children with ADHD, assess glymphatic function via DTI-ALPS, and explore the relationship between NVC and clinical symptoms, executive function, and the ALPS index.

## Materials and methods

### Subjects

Shiyan Taihe Hospital approved this prospective study in accordance with the 1964 Declaration of Helsinki and its later amendments through its institutional review board. Written informed consent was obtained from each participant and their parents.

Between October 2022 and October 2023, we conducted scans on a total of 112 individuals diagnosed with ADHD and 69 healthy controls (HC). Of these, 25 ADHD cases and 11 HC cases were excluded due to incomplete scans. Additionally, 4 ADHD cases and 6 HC cases were excluded because of unusable scans resulting from specific lesions, and 17 ADHD cases and 11 HC cases were excluded due to excessive head movement. Consequently, the study ultimately included 66 participants with ADHD (49 males and 17 females, age range = 6.0 to 14.0 years) and 41 healthy controls (25 males and 16 females, age range = 6.0 to 13.7 years).

The inclusion criteria for the ADHD group were as follows: (1) Children with ADHD, as defined by the criteria outlined in the fifth edition of the Diagnostic and Statistical Manual of Mental Disorders (DSM-V). (2) Right-handed; (3) No contraindication to MRI examination; (4) Any gender, ages 6–14. Patient exclusion criteria included: (1) Other psychiatric diseases. (2) History of use of psychotropic drugs. (3) Organic lesions in the brain. (4) MRI contraindications. HC were enrolled based on the inclusion criteria: (1) No neurological or neuropsychiatric disorders. (2) Other inclusion criteria were similar to those for ADHD participants. Exclusion criteria for HC: (1) psychiatric diseases. (2) the other exclusion criteria were the same as for the ADHD group.

### Clinical characteristics

The Behavior Rating Inventory of Executive Function (BRIEF) [23] and Swanson Nolan and Pelham-IV (SNAP-IV) [24, 25] scales were completed by parents. To obtain information about school-related behaviors, parents were encouraged to consult with teachers when uncertain about specific items, and an experienced clinician also conducted a semi‑structured interview with teachers in cases where parent reports appeared inconsistent with expected school functioning. We also collected demographic data, including age, gender, and years of education, and gathered the approximate average sleep duration of each subject using the parent reporting method. We asked parents to report the average sleep duration in a typical situation. This question did not strictly limit the inquiry to the past week, month, or three months. our data collection was concentrated during the normal academic term (avoiding long holidays), and we did not inquire about illness or travel. While this approach is commonly used in epidemiological surveys. The SNAP-IV was used to assess inattention and hyperactivity/impulsivity symptoms. The BRIEF was used to evaluate executive function abilities among children aged six and older. There are several components of the BRIEF, including the behavioral regulation index (BRI) and the metacognition index (MCI). The BRI includes inhibit, shift, and emotional control, while MCI consists of the plan, initiate, organization, working memory, and monitor. Age and sex-normed T-scores were used for the analysis. Higher SNAP-IV scores indicate more severe clinical symptoms, while higher BRIEF scores indicate greater executive dysfunction.

### Magnetic resonance imaging acquisition

A 3.0-Tesla MRI scanner (Signa Architect, GE Healthcare, Milwaukee, WI, USA) equipped with a 48-channel phased array head coil was used to examine all participants.

The dataset we acquired comprises DTI, rs-fMRI, 3D-pCASL, and 3D-T1 sequence. Parameters used to obtain DTI data are as follows: diffusion encoding was applied in 30 directions with one b-value (b = 1000 s/mm^2^) and one non-diffusion weighted image (b = 0 s/mm^2^), flip angle (FA) = 90^o^, repetition time (TR) = 8819 ms, echo time (TE) = 90.4 ms, field of view (FOV) = 240 mm × 240 mm, matrix = 120 × 120, NEX = 1, slice thickness = 2 mm, slice gap = 0 mm. Following are the parameters used to collect the rs-fMRI data: FA = 90^o^, TR = 2000 ms, TE = 30 ms, FOV = 240 mm × 240 mm, matrix = 80 × 80, slice thickness = 3 mm, slice gap = 0 mm. We addressed the issue of magnetic field non-uniformity by using a uniform field, and we scanned a total of 240 time points in 8 min. 3D-pCASL data were obtained with the following parameters: TR = 4685 ms, TE = 10.7 ms, FOV = 240 mm × 240 mm, slice thickness = 4.0 mm, slice gap = 0 mm, post-labeling delay = 1.5 s. 3D-T1 was obtained with the following parameters: FA = 12^o^, TE = 3.0 ms, TR = 7.4 ms, FOV = 240 mm × 240 mm, matrix = 240 × 240, slice thickness = 1.0 mm without gap.

### rs-fMRI, 3D-pCASL, and NVC processing

Four rs-fMRI metrics were computed using RESTplus: regional homogeneity (ReHo), amplitude of low frequency fluctuation (ALFF), fractional ALFF (fALFF), and degree centrality (DC). These indicators describe four key dimensions of spontaneous neural activity in the brain: local consistency, activity intensity, intensity of activity in specific frequency bands, and the role of global integration hubs, respectively. Analyzing four indicators together allows for a comprehensive assessment of brain region activity, neural coordination, and integration within the brain network, avoiding the limitations of a single metric. This multidimensional approach, combined with CBF analysis, helps explore how neural activity and vascular responses interact. It’s particularly useful for studying ADHD, which involves irregular neural activity, network issues, and neurovascular coupling problems. By merging CBF with BOLD-based indicators, researchers can detect abnormal neural patterns in ADHD and determine if these are linked to cerebral perfusion issues, revealing potential decoupling between neuronal activity and blood flow.

The preprocessing of rs-fMRI followed standard steps: (1) file format conversion; (2) removing the first 10 time points (resulting in 230 time points) to ensure the stability of the initial signal; (3) slice timing correction for the remaining 230 time points. (4) realignment for the head motion correction. The motion correction threshold is set at 2.5° in this study. Motion artifacts can affect data quality, and motion processing is crucial for functional analysis results [26–28]. Furthermore, we computed the mean framewise displacement (FD) for each participant and conducted an inter-group comparison between the HC group and the ADHD group (*z* = -1.788, *P* = 0.074). Therefore, head motion is unlikely to be a confounding factor in our main analyses. (5) reorientation; (6) normalization; (7) smoothing using a Gaussian kernel with a full-width at half-maximum of 6 mm to improve statistical efficiency; (8) detrending; (9) nuisance covariate regression; (10) filtering: the frequency range is 0.01 to 0.08 Hz. Following this, postprocessing is performed to calculate the ALFF, fALFF, ReHo, and DC. Finally, Z-transformed data are chosen for further analysis. CBF preprocessing (SPM12) included linear co-registration to T1, segmentation, normalization to MNI space (2 mm isotropic). Whole-brain mean normalization, and smoothing with an 8 mm FWHM Gaussian kernel. Multiple comparisons were corrected using family wise error (FWE).

NVC calculation (DPABI) quantified four coupling modes (CBF-ALFF, CBF-fALFF, CBF-ReHo, CBF-DC). CBF maps were resampled to 3 mm to match rs-fMRI resolution. For each participant, voxel-wise correlation coefficients between CBF and each rs-fMRI metric were computed within a gray matter mask. The multiple comparison correction uses the FWE method. We conducted a coupling analysis across 90 brain regions (AAL_90 atlas) based on coupling indicators that showed statistically significant differences in the whole-brain NVC analysis, aiming to identify specific brain regions exhibiting variations in NVC values. The AAL_90 atlas was adopted for regional analyses due to its widespread use in ADHD research, balanced anatomical coverage, and compatibility with multimodal registration in MNI space. Following the extraction of NVC values, intergroup comparisons were performed, and the results were corrected for multiple comparisons using the false discovery rate (FDR) method.

### Diffusion tensor data and ALPS processing

The FMRIB Software Library (FSL) was employed for post-processing DTI data, including correcting eddy current distortions, addressing motion artifacts, and performing skull stripping. Using the FSL software, the diffusion rate images of the projection fibers, association fibers, and subcortical fibers along the x, y, and z directions were generated. Two neuroimaging experts, one with 10 years of experience and the other with 8 years in the field, who were blind to the original data, utilized fsleyes software to map the regions of interest (ROI). Given that all participants in this study were right-handed, we defined the ROI as a sphere with a radius of 2.5 mm centered at the level of the left lateral ventricle body [14]. The delineated area was located within the walking zone of the medullary vein along the vertical wall of the ventricle, as the orientation of the perivascular space aligned with that of the medullary vein. The ROIs for projection fibers, and association fibers were positioned on a consistent horizontal plane. The DPABI toolkit was utilized to extract the values. These values include Dxx_proj, Dyy_proj, Dzz_proj, Dxx_associ, Dyy_associ, and Dzz_associ, and the ALPS index was calculated using the designated formula, which was corrected by FWE multiple comparison.

$$\:\mathrm{A}\mathrm{L}\mathrm{P}\mathrm{S}\:\mathrm{i}\mathrm{n}\mathrm{d}\mathrm{e}\mathrm{x}=\frac{\mathrm{m}\mathrm{e}\mathrm{a}\mathrm{n}\:(\mathrm{D}\mathrm{x}\mathrm{x}\_\mathrm{p}\mathrm{r}\mathrm{o}\mathrm{j},\:\mathrm{D}\mathrm{x}\mathrm{x}\_\mathrm{a}\mathrm{s}\mathrm{s}\mathrm{o}\mathrm{c}\mathrm{i})}{\mathrm{m}\mathrm{e}\mathrm{a}\mathrm{n}\:(\mathrm{D}\mathrm{y}\mathrm{y}\_\mathrm{p}\mathrm{r}\mathrm{o}\mathrm{j},\:\mathrm{D}\mathrm{z}\mathrm{z}\_\mathrm{a}\mathrm{s}\mathrm{s}\mathrm{o}\mathrm{c}\mathrm{i})}$$

### Statistical analysis

A Statistical Package for the Social Sciences (SPSS) version 25.0 was used to analyze clinical data, the ALPS index, and NVC for the ADHD and HC groups. For data that followed a normal distribution, independent sample *t*-tests were employed, and Mann-Whitney U-tests were used for variables that did not follow a normal distribution. The enumerated data were analyzed using Chi-squared tests. Based on our integrated hypothesis, we structured the analyses into two hypothesis‑driven steps.

We postulated that children with ADHD exhibit abnormal NVC and glymphatic function. To evaluate this hypothesis, two-sample *t*-test were conducted to identify differences between the ADHD and HC groups in the four global NVC metrics (CBF-ALFF, CBF-fALFF, CBF-ReHo, and CBF-DC), with corrections for multiple comparisons applied using FWE (*P*-FWE < 0.05/4). To identify specific brain regions showing NVC differences, we performed regional analysis using the AAL_90 atlas (90 regions). Two‑sample *t*‑tests were applied for each region, and the FDR was used for correction (*P*-FDR < 0.05). Additionally, we compared the four rs‑fMRI indicators (ALFF, fALFF, ReHo, DC) and CBF between groups using a general linear model with age, sex, and education as covariates (*P*‑FDR < 0.05). The comparison of the ALPS index was performed using the two-sample independent *t*-test. The Intraclass Correlation Coefficient (ICC) was utilized to evaluate the agreement between measurements obtained by different observers in the ALPS assessment.

Moreover, we hypothesized that impaired NVC function and glymphatic dysfunction are linked to the core clinical manifestations of ADHD, particularly executive deficits. To investigate this, partial correlation analyses were used to explore the relationships among clinical data, executive function measures, ALPS index, and abnormal NVC metrics in the ADHD group. Receiver operating characteristic (ROC) curve analysis was performed to evaluate the ability to diagnose ADHD. A combined diagnostic model was established using logistic regression (included HC participants in model construction), and its performance was evaluated by five-fold cross-validation.

## Results

### Demographic and clinical characteristics

As shown in Table 1, the group differences in gender (*x*^*2*^ = 2.087, *P* = 0.149), age (*z* = -0.773, *P* = 0.439), and education years (*z* = -1.432, *P* = 0.152) are not significant. ADHD group participants had shorter sleep duration than HC participants, and ADHD group patients performed worse on attention, hyperactivity, and BRIEF tests (*P* < 0.001).

**Table 1:** Demographic, clinical data and executive function scores of participants

Characteristics	ADHD (*n* = 66)	HC (*n* = 41)	Statistical value (x^2^ /t/z)	*P*-value
Gender(M/F)	49/17	25/16	2.087	0.149^c^
Age	9.3(8, 10.6)	10(8, 12)	-0.773	0.439 ^b^
Education years	3(2, 5)	4(2, 6)	-1.432	0.152^b^
Sleep duration (hour)	7.7(7.3, 8.0)	8.2(8.0, 8.4)	-5.454	**< 0.001** ^b*^
Attention Defect Score	1.78(1.66, 2.00)	0.88(0.55, 1.16)	-8.675	**< 0.001** ^b*^
Hyperactivity Score	1.60 ± 0.38	0.54 ± 0.36	-14.211	**< 0.001** ^a*^
Inhibit	58(51, 66)	45(41, 49.5)	-6.347	**< 0.001** ^b*^
Shift	51(47, 59)	45(40, 50)	-4.003	**0.001** ^b*^
Emotional Control	53(47, 60.5)	47(39, 51)	-3.832	**0.001** ^b*^
Initiate	62.5(54.5, 69)	46(40.5, 55.5)	-6.011	**< 0.001** ^b*^
Working Memory	64.19 ± 10.95	48.41 ± 7.19	-8.189	**< 0.001** ^a*^
Plan	64.80 ± 7.04	49.63 ± 8.06	-10.241	**< 0.001** ^a*^
Organization	64(57, 67)	46(37, 53.5)	-6.563	**< 0.001** ^b*^
Monitor	66.5(59.75, 75)	46(40, 55.5)	-6.866	**< 0.001** ^b*^
Behavioral Regulation Index	55(50, 62)	46(40, 50.5)	-5.707	**< 0.001** ^b*^
Metacognitive Index	66.53 ± 5.19	48.17 ± 8.71	-13.669	**< 0.001** ^a*^

Characteristics of demographic and clinical data and executive function scores of participants. ^*a*^Independent samples t–test (*t*), expressed as mean ± standard deviation. ^*b*^Mann–Whitney U-test (*z*), expressed by median (quartile). ^*c*^Chi-square test (*x*^*2*^). ^*^P-values in bold show significant differences (^*^P < 0.05). ADHD: attention-deficit/hyperactivity disorder, HC: health control

### ALPS results

**Fig. 1:**
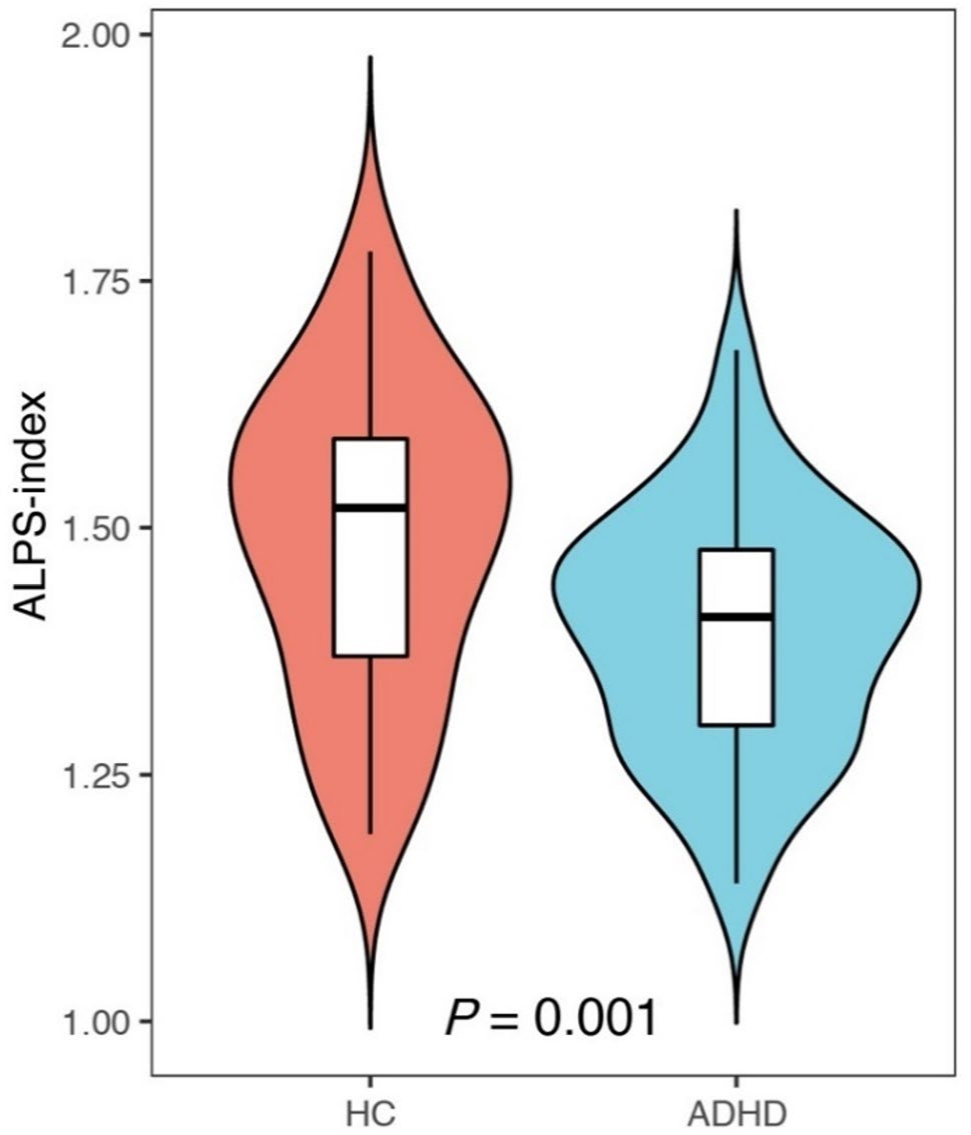
The mean ALPS-index value between HC and ADHD group

The ICC for the inter-observer ALPS index is 0.902, 95% CI: (0.773, 0.960). Compared with HC (1.48 ± 0.15), ADHD patients had a lower ALPS index (1.39 ± 0.12) (*P* = 0.001) (Fig. 1).

### rs-fMRI, 3D-pCASL, and NVC results

**Fig. 2:**
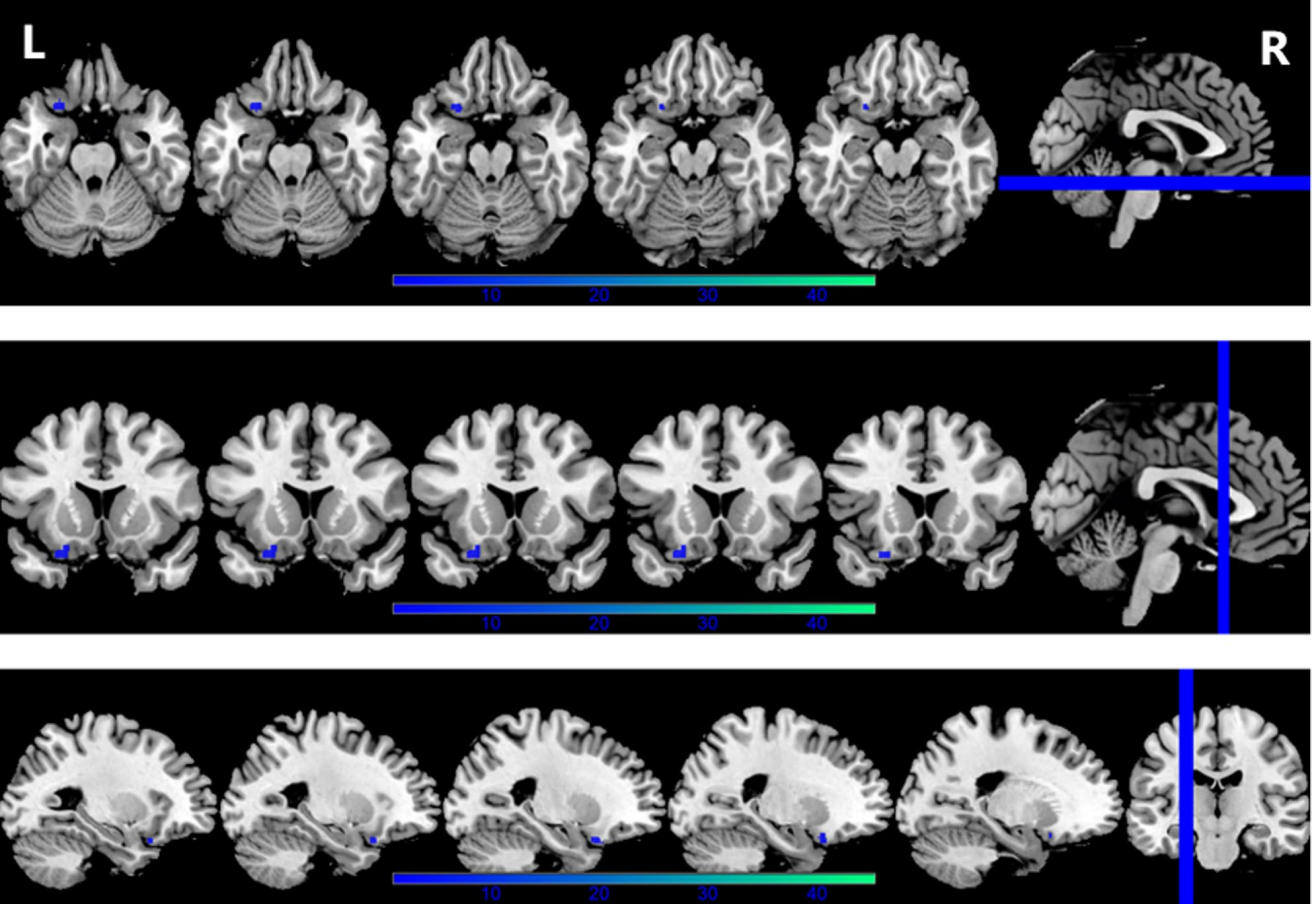
Between-group differences of CBF. Compared with HC, ADHD patients showed significantly decreased CBF in the left inferior frontal gyrus-orbital part. Blue color represents regions that CBF values have decreased. ADHD: attention-deficit/hyperactivity disorder, HC: health control, CBF: cerebral blood flow

**Fig. 3:**
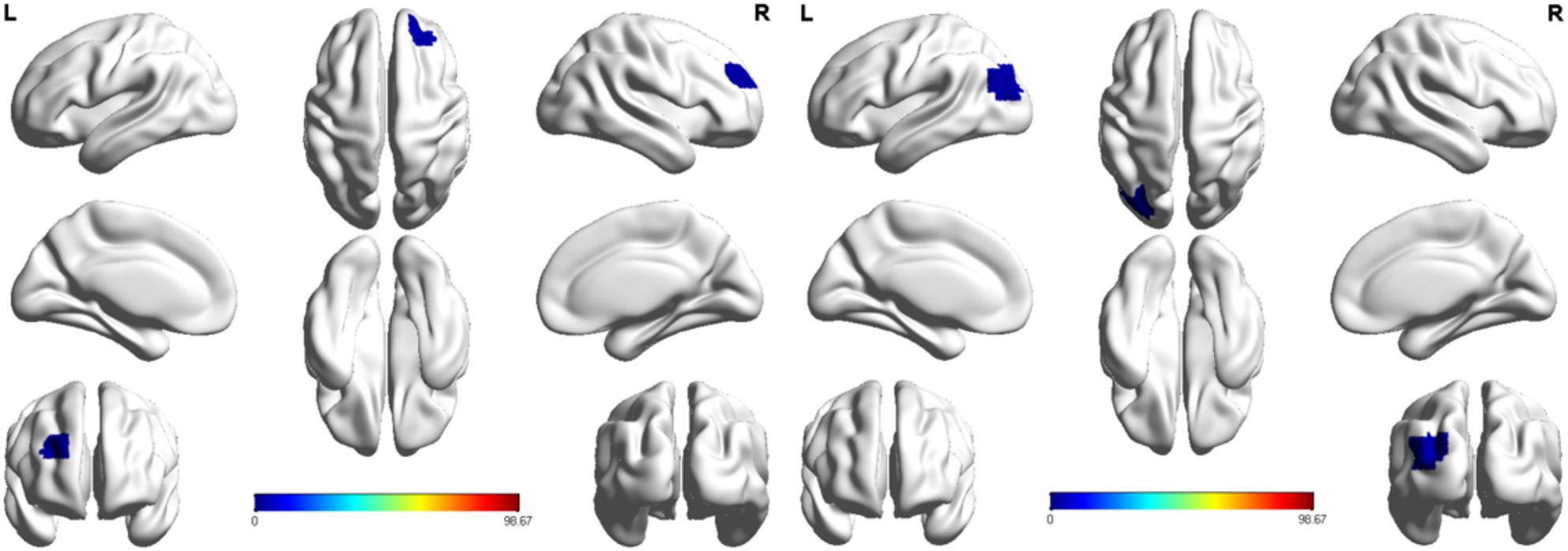
Between-group differences of fALFF. Compared with HC, ADHD patients showed significantly decreased fALFF in the right middle frontal gyrus and left middle occipital gyrus. Blue color represents regions that fALFF values have decreased. ADHD: attention-deficit/hyperactivity disorder, HC: health control, fALFF: fractional amplitude of low frequency fluctuation

**Fig. 4:**
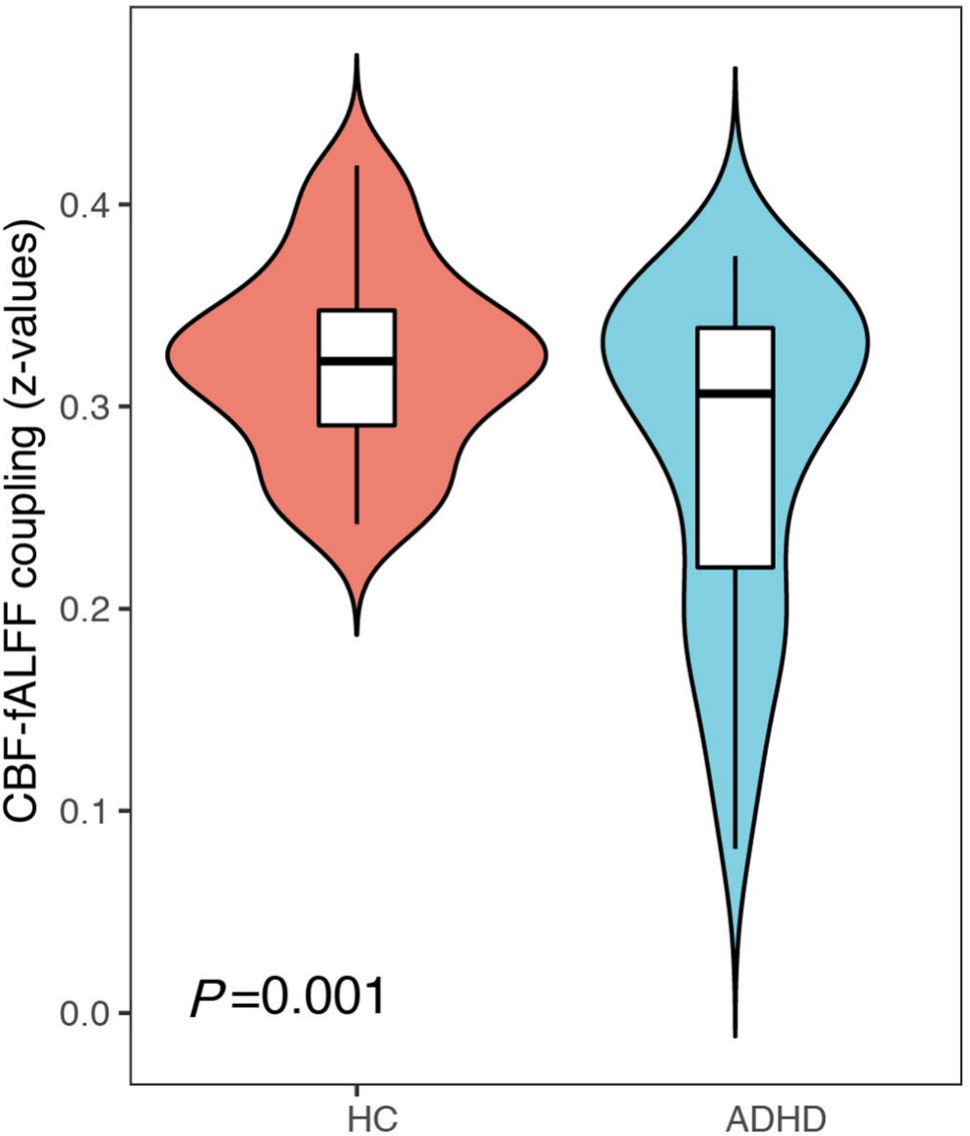
Between-group differences of global CBF-fALFF coupling. Compared with HC, ADHD patients showed significantly decreased CBF-fALFF coupling. ADHD: attention-deficit/hyperactivity disorder, HC: health control

**Fig. 5:**
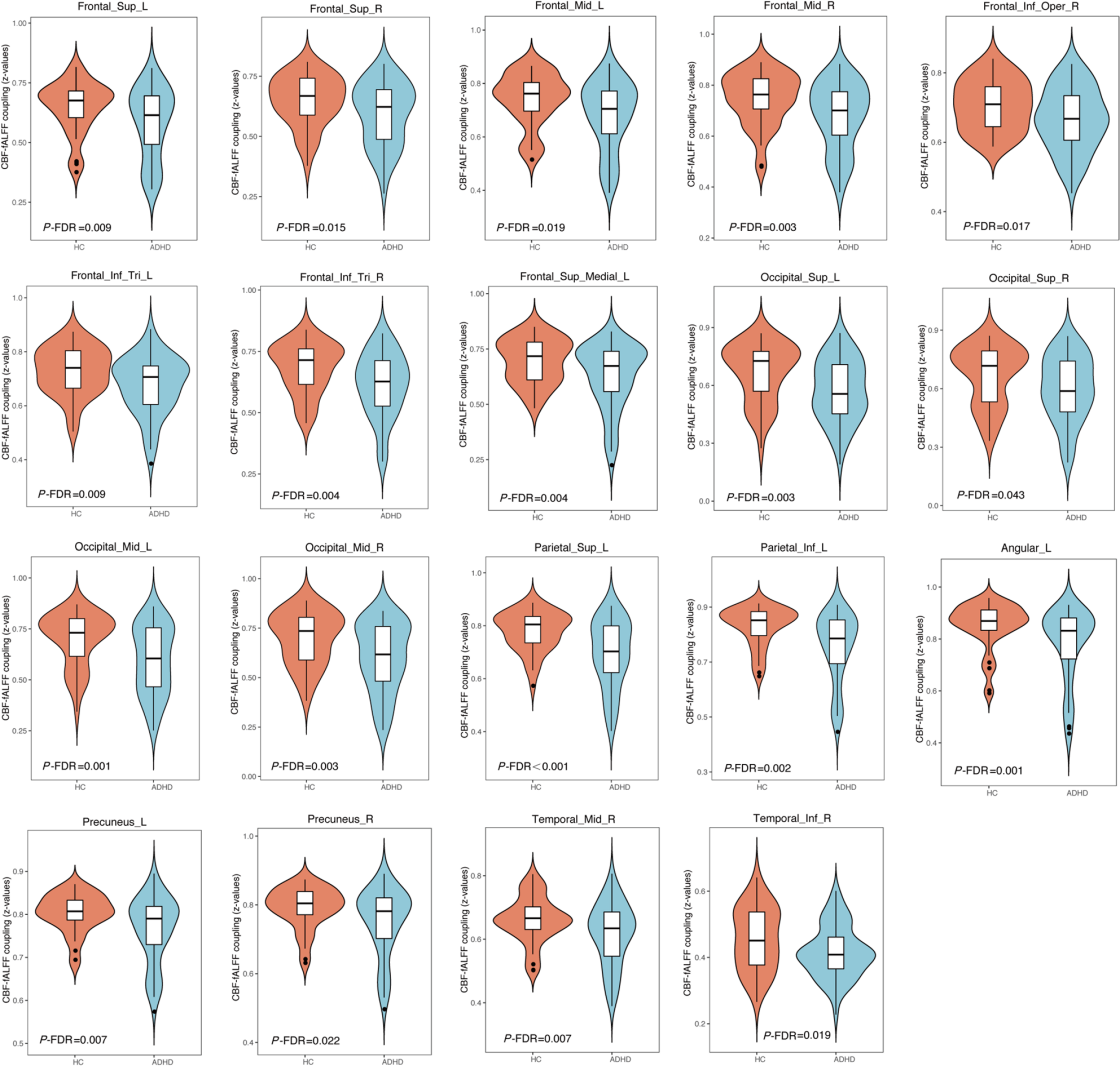
Between-group differences of regional CBF-fALFF coupling. Compared with HC, ADHD patients showed significantly decreased CBF-fALFF coupling in 19 brain regions. Frontal_Sup_L: left superior frontal gyrus-dorsolateral, Frontal_Sup_R: right superior frontal gyrus-dorsolateral, Frontal_Mid_L: left middle frontal gyrus, Frontal_Mid_R: right middle frontal gyrus, Frontal_Inf_Oper_R: right middle frontal gyrus-orbital part, Frontal_Inf_Tri_L: left inferior frontal gyrus-triangular part, Frontal_Inf_Tri_R: right inferior frontal gyrus-triangular part, Frontal_Sup_Medial_L: left superior frontal gyrus-medial, Occipital_Sup_L: left superior occipital gyrus, Occipital_Sup_R: right superior occipital gyrus, Occipital_Mid_L: left middle occipital gyrus, Occipital_Mid_R: right middle occipital gyrus, Parietal_Sup_L: left superior parietal gyrus, Parietal_Inf_L: left inferior parietal-supramarginal and angular gyri, Angular_L: Precuneus_L: left angular gyrus, left precuneus, Precuneus_R: right precuneus, Temporal_Mid_R: right middle temporal gyrus, Temporal_Inf_R: right inferior temporal gyrus. ADHD: attention-deficit/hyperactivity disorder, HC: health control

Among the results that passed multiple comparison correction, the FWE cluster size threshold for the CBF analysis is 36, and the FWE cluster size threshold for the fALFF analysis is 32. A voxel-level analysis shows that there was a decrease CBF in the left inferior frontal gyrus-orbital part (Frontal-Infra-Orb-L) (Peak intensity = 3.7955, Fig. 2; Table 2), as well as a decrease in fALFF in the left middle occipital gyrus (Occipital_Mid_L) (Peak intensity = 4.0315) and right middle frontal gyrus (Frontal_Mid_R) (Peak intensity = 4.1196) in ADHD group (Fig. 3; Table 2). ADHD patients had significantly reduced global CBF-fALFF coupling (*t* = 3.315, *P* = 0.001) (Fig. 4; Table 3), however, no significant difference was found in CBF-ALFF, CBF-ReHo, and CBF-DC coupling (Table 3). At the regional level, the ADHD group had decreased CBF-fALFF in 19 brain regions of AAL_90 (Fig. 5; Table 4), which included left superior frontal gyrus-dorsolateral (Frontal_Sup_L) (*t* = 2.699, *P*-FDR = 0.009), right superior frontal gyrus-dorsolateral (Frontal_Sup_R) (*t* = 2.519, *P*-FDR = 0.015), left middle frontal gyrus (Frontal_Mid_L) (*t* = 2.449, *P*-FDR = 0.019), right middle frontal gyrus (Frontal_Mid_R) (*t* = 3.059, *P*-FDR = 0.003), right middle frontal gyrus-orbital part (Frontal_Inf_Oper_R) (*t* = 2.494, *P*-FDR = 0.017), left inferior frontal gyrus-triangular part (Frontal_Inf_Tri_L) (*t* = 2.693, *P*-FDR = 0.009), right inferior frontal gyrus-triangular part (Frontal_Inf_Tri_R) (*t* = 2.938, *P*-FDR = 0.004), left superior frontal gyrus-medial (Frontal_Sup_Medial_L) (*t* = 2.965, *P*-FDR = 0.004), left superior occipital gyrus (Occipital_Sup_L) (*t* = 3.038, *P*-FDR = 0.003), right superior occipital gyrus (Occipital_Sup_R) (*t* = 2.148, *P*-FDR = 0.043), left middle occipital gyrus (Occipital_Mid_L) (*t* = 3.586, *P*-FDR = 0.001), right middle occipital gyrus (Occipital_Mid_R) (*t* = 3.037, *P*-FDR = 0.003), left superior parietal gyrus (Parietal_Sup_L) (*t* = 3.647, *P*-FDR < 0.001), left inferior parietal-supramarginal and angular gyri (Parietal_Inf_L) (*t* = 3.138, *P*-FDR = 0.002), left angular gyrus (Angular_L) (*t* = 3.346, *P*-FDR = 0.001), left precuneus (Precuneus_L) (*t* = 2.803, *P*-FDR = 0.007), right precuneus (Precuneus_R) (*t* = 2.404, *P*-FDR = 0.022), right middle temporal gyrus (Temporal_Mid_R) (*t* = 2.793, *P*-FDR = 0.007), right inferior temporal gyrus (Temporal_Inf_R) (*t* = 2.460, *P*-FDR = 0.019).

**Table 2:** Brain regions with significant differences between ADHD and HC in CBF and fALFF

Parameter	Cluster size	Peak MNI coordinate			Peak MNI region	Peak intensity	Cluster region
		x	y	z			
CBF	36	-24	16	-24	Frontal_Inf_Orb_L	3.7955	Frontal_Inf_Orb_L
fALFF-cluster 1	41	-27	-78	27	Occipital_Mid_L	4.0315	Occipital_Mid_L
fALFF-cluster 2	32	27	51	30	Frontal_Mid_R	4.1196	Frontal_Mid_R

ADHD: attention-deficit/hyperactivity disorder, HC: health control, CBF: cerebral blood flow, fALFF: fractional amplitude of low frequency fluctuation, Frontal_Inf_Orb_L: left inferior frontal gyrus-orbital part, Occipital_Mid_L: left middle occipital gyrus, Frontal_Mid_R: right middle frontal gyrus

**Table 3:** Between-group differences of global NVC

NVC	ADHD (*n* = 66)	HC (*n* = 41)	Statistical value (t)	*P*-value
CBF-ALFF	0.163 ± 0.046	0.178 ± 0.035	1.738	0.085
CBF-fALFF	0.277 ± 0.079	0.322 ± 0.047	3.315	**0.001** ^*^
CBF-ReHo	0.301 ± 0.042	0.302 ± 0.043	0.131	0.896
CBF-DC	0.233 ± 0.072	0.241 ± 0.070	0.565	0.573

The outcomes of cross-voxel level comparisons between the ADHD group and the HC group across four NVC modalities. NVC: neurovascular coupling, ADHD: attention-deficit/hyperactivity disorder, HC: health control. ^*^P-values in bold show significant differences (^*^P < 0.05/4)

**Table 4:** Between-group differences of rgional NVC

Region	ADHD	HC	Statistical value (t)	*P*-value	*P*-FDR
Frontal_Sup_L	0.580 ± 0.154	0.648 ± 0.107	2.699	0.008	**0.009**
Frontal_Sup_R	0.591 ± 0.130	0.649 ± 0.104	2.519	0.013	**0.015**
Frontal_Mid_L	0.675 ± 0.137	0.737 ± 0.106	2.449	0.016	**0.019**
Frontal_Mid_R	0.677 ± 0.131	0.746 ± 0.102	3.059	0.003	**0.003**
Frontal_Inf_Oper_R	0.660 ± 0.104	0.706 ± 0.073	2.494	0.014	**0.017**
Frontal_Inf_Tri_L	0.675 ± 0.105	0.728 ± 0.088	2.693	0.008	**0.009**
Frontal_Inf_Tri_R	0.612 ± 0.130	0.682 ± 0.101	2.938	0.004	**0.004**
Frontal_Sup_Medial_L	0.625 ± 0.145	0.696 ± 0.099	2.965	0.004	**0.004**
Occipital_Sup_L	0.574 ± 0.160	0.668 ± 0.149	3.038	0.003	**0.003**
Occipital_Sup_R	0.601 ± 0.167	0.670 ± 0.150	2.148	0.034	**0.043**
Occipital_Mid_L	0.597 ± 0.166	0.700 ± 0.129	3.586	0.001	**0.001**
Occipital_Mid_R	0.603 ± 0.173	0.699 ± 0.132	3.037	0.003	**0.003**
Parietal_Sup_L	0.682 ± 0.145	0.769 ± 0.100	3.647	<0.001	**<0.001**
Parietal_Inf_L	0.741 ± 0.153	0.821 ± 0.111	3.138	0.002	**0.002**
Angular_L	0.769 ± 0.169	0.852 ± 0.088	3.346	0.001	**0.001**
Precuneus_L	0.757 ± 0.091	0.798 ± 0.060	2.803	0.006	**0.007**
Precuneus_R	0.751 ± 0.097	0.789 ± 0.065	2.404	0.018	**0.022**
Temporal_Mid_R	0.615 ± 0.097	0.661 ± 0.072	2.793	0.006	**0.007**
Temporal_Inf_R	0.413 ± 0.084	0.456 ± 0.094	2.460	0.016	**0.019**

Brain regions with significant differences between ADHD and HC in rgional NVC. Frontal_Sup_L: left superior frontal gyrus-dorsolateral, Frontal_Sup_R: right superior frontal gyrus-dorsolateral, Frontal_Mid_L: left middle frontal gyrus, Frontal_Mid_R: right middle frontal gyrus, Frontal_Inf_Oper_R: right middle frontal gyrus-orbital part, Frontal_Inf_Tri_L: left inferior frontal gyrus-triangular part, Frontal_Inf_Tri_R: right inferior frontal gyrus-triangular part, Frontal_Sup_Medial_L: left superior frontal gyrus-medial, Occipital_Sup_L: left superior occipital gyrus, Occipital_Sup_R: right superior occipital gyrus, Occipital_Mid_L: left middle occipital gyrus, Occipital_Mid_R: right middle occipital gyrus, Parietal_Sup_L: left superior parietal gyrus, Parietal_Inf_L: left inferior parietal-supramarginal and angular gyri, Angular_L: Precuneus_L: left angular gyrus, left precuneus, Precuneus_R: right precuneus, Temporal_Mid_R: right middle temporal gyrus, Temporal_Inf_R: right inferior temporal gyrus. NVC: neurovascular coupling, ADHD: attention-deficit/hyperactivity disorder, HC: health control. *p*-FDR in bold show significant differences (*P*-FDR < 0.05)

### Correlation and ROC results

**Fig. 6:**
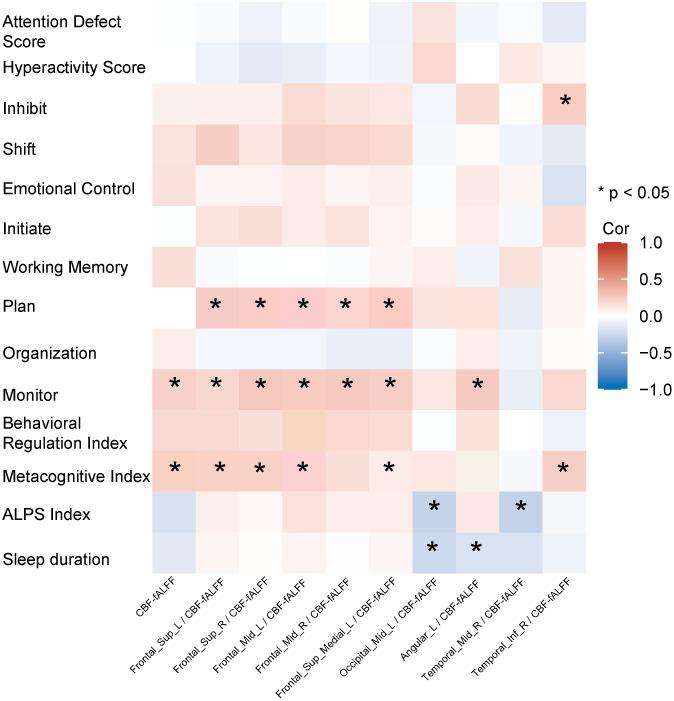
A heatmap showing the correlation between CBF/fALFF values and clinical data, as well as the ALPS index. Color bar on the right side displays the value of the correlation coefficient (higher from − 1 to 1, and from blue to red). *P < 0.05. Frontal_Sup_L: left superior frontal gyrus-dorsolateral, Frontal_Sup_R: right superior frontal gyrus-dorsolateral, Frontal_Mid_L: left middle frontal gyrus, Frontal_Mid_R: right middle frontal gyrus, Frontal_Sup_Medial_L: left superior frontal gyrus-medial, Occipital_Mid_L: left middle occipital gyrus, Angular_L: left angular gyrus, Temporal_Mid_R: right middle temporal gyrus, Temporal_Inf_R: right inferior temporal gyrus

**Fig. 7:**
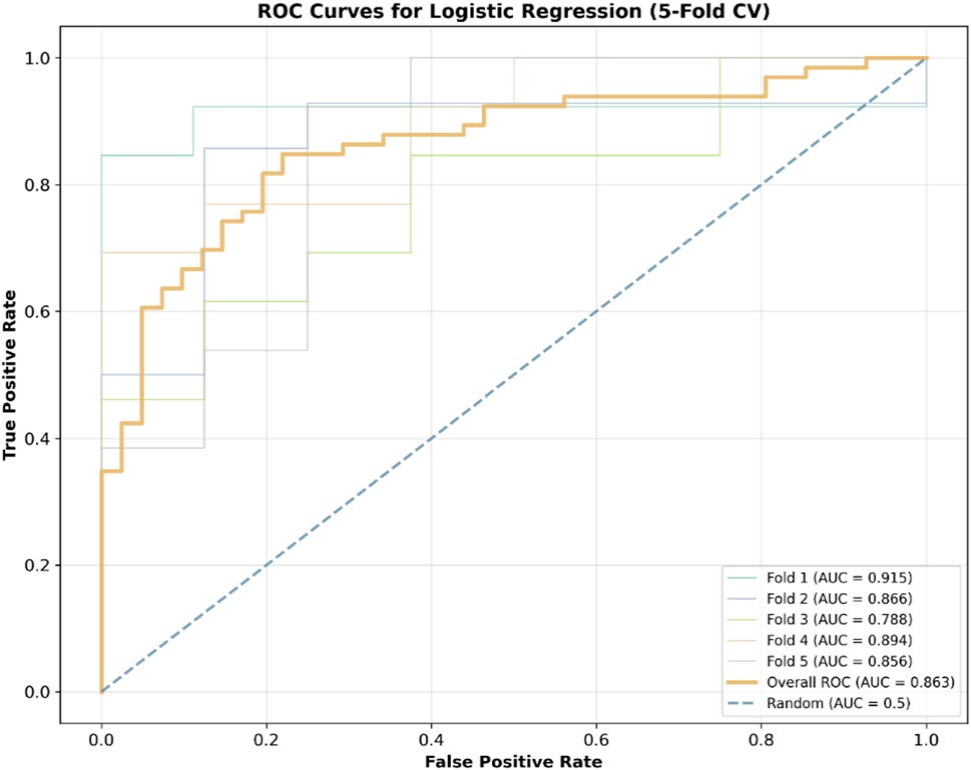
The ROC analysis indicated good diagnostic efficiency in the combined model with five-fold cross-validation, with an AUC of 0.863, corresponding to a sensitivity of 0.864 and a specificity of 0.683. ROC: receiver operating characteristic, AUC: area under the curve, CV: cross-validation

The results of the partial correlation analysis are presented in Fig. 6. At the whole-brain gray matter level, the CBF-fALFF coupling value showed a significant positive correlation with monitor (*r* = 0.281, *P* = 0.026) and the metacognitive index (*r* = 0.268, *P* = 0.034). In the Frontal_Sup_L, CBF-fALFF coupling was positively associated with plan (*r* = 0.263, *P* = 0.037), monitor (*r* = 0.258, *P* = 0.041), and the metacognitive index (*r* = 0.275, *P* = 0.029). Similarly, in the Frontal_Sup_R, significant positive correlations were observed with plan (*r* = 0.309, *P* = 0.014), monitor (*r* = 0.312, *P* = 0.013), and the metacognitive index (*r* = 0.297, *P* = 0.018). In the Frontal_Mid_L, CBF-fALFF coupling was positively correlated with plan (*r* = 0.257, *P* = 0.042), monitor (*r* = 0.286, *P* = 0.023), and the metacognitive index (*r* = 0.292, *P* = 0.020). In the Frontal_Mid_R, significant positive correlations were found with plan (*r* = 0.285, *P* = 0.023) and monitor (*r* = 0.343, *P* = 0.006). The Frontal_Sup_Medial_L exhibited positive associations between CBF-fALFF coupling and plan (*r* = 0.255, *P* = 0.044), monitor (*r* = 0.336, *P* = 0.007), and the metacognitive index (*r* = 0.298, *P* = 0.018). In the Occipital_Mid_L, CBF-fALFF coupling was negatively correlated with the ALPS index (*r* = -0.275, *P* = 0.029) and sleep duration (*r* = -0.255, *P* = 0.043). The Angular_L showed a positive correlation with monitor (*r* = 0.265, *P* = 0.036) and a negative correlation with sleep duration (*r* = -0.251, *P* = 0.047). In the Temporal_Mid_R, CBF-fALFF coupling was negatively correlated with the ALPS index (*r* = -0.289, *P* = 0.021). Finally, in the Temporal_Inf_R, CBF-fALFF coupling was positively correlated with inhibition (*r* = 0.262, *P* = 0.038) and metacognition (*r* = 0.277, *P* = 0.028). ROC analysis of the combined model yielded an AUC of 0.863 (sensitivity 0.864, specificity 0.683) with five-fold cross-validation, 95% CI: (0.790, 0.926) (Fig. 7).

## Discussion

The human brain is in a constant state of metabolic activity; it relies on neurovascular units to precisely regulate blood flow to support the balance between neural activity and metabolic demands [29]. NVC is crucial for maintaining this process [30]. Our direct findings show that CBF-fALFF coupling is significantly reduced in children with ADHD compared to healthy controls, with the reduction primarily localized to frontal, parietal, occipital, and temporal lobes.

In the ADHD group, fALFF values were lower in the right middle frontal gyrus and left middle occipital gyrus. Consistently, a reduction in CBF-fALFF coupling was observed in these two regions. Importantly, no significant group difference in CBF was detected in these areas. These results directly indicate that the observed reduction in NVC in these brain regions is primarily driven by fALFF changes rather than by alterations in CBF. One plausible interpretation is that lower fALFF reflects reduced spontaneous local neuronal activity and decreased neuronal firing intensity. The resulting mismatch between neural activity and cerebral blood flow may contribute to NVC dysfunction. This dysfunction may cause reduced blood perfusion, affect myelin formation and neural signal transduction, and ultimately aggravate local brain dysfunction and clinical symptoms [31, 32]. However, the directionality and long-term consequences remain speculative based on our cross-sectional data, this hypothesis requires direct longitudinal or interventional validation.

However, we observed a paradoxical within-group finding: although average NVC strength in specific ADHD regions was lower than in healthy controls, within the ADHD group, higher NVC strength correlated with poorer executive function performance, a reduced ALPS index, and worse sleep quality. These correlations are directly supported by our data and survived correction for multiple comparisons. One interpretation, which remains speculative given the cross-sectional design, is that neurovascular abnormalities in ADHD may reflect not merely a straightforward functional decline but rather a complex regulatory dysfunction that possibly involves both hyporeactive and hyperreactive patterns. Consistent with this view, the observed within-group paradoxical correlation suggests that NVC does not follow a simple linear “higher is better” relationship. We hypothesize that optimal NVC may operate within a specific physiological range, and deviations in either direction could indicate neural dysfunction. However, due to the modest sample size and lack of ADHD subtype distinction, our findings on heterogeneity should be considered hypothesis-generating. Future studies with larger, well-stratified samples are needed to test whether primary hyporeactive and compensatory over-coupling patterns exist and whether they carry distinct clinical implications.

The following interpretation is purely speculative and intended to generate hypotheses for future testing. It is possible that in ADHD, the efficiency of fundamental cognitive networks is already compromised. The brain might then engage excessive, non-specific neural activity in alternative regions or via atypical patterns, leading to heightened vascular responses. This hypothetical “high-energy, low-efficiency over-coupling” would not facilitate effective cognition and could instead indicate deficient neurovascular regulatory capacity. Conversely, the ability to maintain relatively low vascular responses might reflect preserved brain homeostasis and neurovascular reserve. If this speculative model holds, then factors that support metabolic waste clearance and adequate sleep could stabilize neuronal-vascular function, reducing the need for exaggerated responses and thus manifesting as lower NVC. By contrast, poorer ALPS function might increase metabolic stress, prompting more pronounced and unstable vascular responses, thereby manifesting as increased NVC. We emphasize that these mechanistic propositions are not directly testable with our current cross-sectional data and require longitudinal, interventional, or animal model studies.

The central executive network (CEN) is crucial for the performance of executive functions [33]. Some higher cognitive functions, including working memory and attention, are mediated by CEN [34, 35]. The precuneus, subparietal lobule, and inferior temporal gyrus are known components of DMN [36–38]. Previous studies have shown that the functional connectivity of ADHD patients in the prefrontal and parietal regions is significantly reduced, especially in networks involving executive function and attention control [39, 40]. In addition, the DMN activity in children with ADHD is excessive, and this excessive activity is closely related to the impaired functional connection between the DMN and the executive control network during task performance [41, 42]. Our findings both converge with and diverge from previous ADHD neuroimaging studies. Compared with these studies, our research explores the role of NVC in ADHD, although it does not focus on the connection patterns of brain regions, but our research found that the brain regions with NVC dysfunction are mostly located in the CEN and the DMN. Furthermore, our research found that executive function performance in the ADHD group was significantly worse than that of the HC group. NVC dysfunction in the frontal-parietal region, particularly in the right middle frontal gyrus, is significantly correlated with impairments in executive functions, such as plan and monitor. This finding supports the existence of executive dysfunction in individuals with ADHD and suggests a potential link between executive impairments and NVC dysfunction.

Previous studies have found that the DMN in patients with ADHD is abnormal [43–45]. Our research has revealed a reduction in NVC at key nodes of the DMN, including the bilateral precuneus, left angular gyrus, and right inferior temporal gyrus. Additionally, the dysfunction in CBF-fALFF coupling in the left angular gyrus and right inferior temporal gyrus was positively correlated with impairments in monitor and inhibit of executive function. Based on these research findings, we speculate that the NVC dysfunction may result in impaired connectivity within the network, thereby causing a series of clinical and functional disorders. Future research should prioritize a systematic investigation of network connections, with particular emphasis on elucidating their underlying structural and functional interrelationships.

Prior studies have suggested that NVC contributes not only to metabolite supply but also to metabolic waste [16], raising the possibility that NVC status may relate to glymphatic function. Our data provide correlational support for this hypothesis: we found that NVC dysfunction in the left middle occipital gyrus, right middle temporal gyrus, and right inferior temporal gyrus was significantly correlated with both the ALPS index and sleep duration. However, given the cross-sectional design, we cannot determine the direction of these associations: whether NVC dysfunction leads to reduced glymphatic clearance, whether impaired glymphatic function worsens NVC, or whether both are driven by a third factor. Therefore, we interpret these correlations as preliminary evidence of an association between NVC and glymphatic/sleep measures in ADHD, rather than as evidence of causal interaction.

In conclusion, our research indicates that NVC abnormalities mainly affect the brain networks related to executive control and attention regulation. NVC abnormalities are not just statistical differences; they are associated with the core clinical symptoms and glymphatic function in children with ADHD. Our results suggest that there might be an “optimal range” for NVC. Deviating from this range (whether too low or too high) could be pathological. Future studies need to be conducted on large scale, longitudinal, and subtype-stratified cohorts to perform individualized assessments of ADHD.

### Limitation

Several limitations should be acknowledgment. First, the sample size limited the statistical power to detect small-to-moderate effects; thus, positive findings may be overestimated, while null findings remain inconclusive. Larger cohorts are needed for replication. Second, the absence of subtype analyses may mask within-group heterogeneity; future studies should collect sufficient samples for subgroup comparisons. Third, intelligence quotient data were not collected, precluding adjustment for cognitive confounds; it should be included as a covariate in future research. Fourth, the single-center cross-sectional design precludes causal inference; multi-center longitudinal designs are required. Fifth, age-related changes in NVC were not examined, potentially confounding the results by neurodevelopmental trajectories; future longitudinal studies should disentangle age effects. Sixth, direct glymphatic measures were lacking, making our interpretation of the NVC–glymphatic interaction speculative; future work should incorporate direct imaging. Seventh, the AAL_90 atlas, which is anatomically based, may underestimate effects due to functional heterogeneity within regions; future studies should use functional parcellations. Eighth, sleep duration measurement was limited, introducing recall bias; future studies should utilize device-based tracking in conjunction with sleep logs.

## Conclusions

Children with ADHD exhibited reduced CBF-fALFF coupling in the frontal, parietal, occipital, and temporal regions, and this reduction was associated with poorer executive function performance. Additionally, lower coupling in specific regions correlated with a reduced ALPS index and shorter sleep duration. It suggests that neurovascular and glymphatic perspectives may provide additional avenues for understanding ADHD-related brain dysfunction.

## Data Availability

The datasets used and analyzed during the current study are available from the corresponding author on reasonable request.

## Abbreviations

NVC: Neurovascular coupling
ADHD: Attention-deficit/hyperactivity disorder
HC: Healthy controls
DTI: Diffusion tensor imaging
ALPS: Along the perivascular space
ALFF: Amplitude of low frequency fluctuation
fALFF: Fractional ALFF
ReHo: Regional homogeneity
DC: Degree centrality
3D-pCASL: Three-dimensional pseudo-continuous arterial spin labeling
CBF: Cerebral blood flow
CEN: Central executive network
DMN: Default mode network
NVC: Neurovascular coupling
BRI: Behavioral regulation index
MCI: Metacognition index
ICC: Interclass Correlation Coefficient
ROC: Receiver operating characteristic
AUC: Area under the curve
Frontal_Sup_L: Left superior frontal gyrus-dorsolateral
Frontal_Sup_R: Right superior frontal gyrus-dorsolateral
Frontal_Mid_L: Left middle frontal gyrus
Frontal_Mid_R: Right middle frontal gyrus
Frontal_Inf_Oper_R: Right middle frontal gyrus-orbital part
Frontal_Inf_Tri_L: Left inferior frontal gyrus-triangular part
Frontal_Inf_Tri_R: Right inferior frontal gyrus-triangular part
Frontal_Sup_Medial_L: Left superior frontal gyrus-medial
Occipital_Sup_L: Left superior occipital gyrus
Occipital_Sup_R: Right superior occipital gyrus
Occipital_Mid_L: Left middle occipital gyrus
Occipital_Mid_R: Right middle occipital gyrus
Parietal_Sup_L: Left superior parietal gyrus
Parietal_Inf_L: Left inferior parietal-supramarginal and angular gyri
Angular_L: Left angular gyrus
Precuneus_L: Left precuneus
Precuneus_R: Right precuneus
Temporal_Mid_R: Right middle temporal gyrus
Temporal_Inf_R: Right inferior temporal gyrus
